# Dot1l expression predicts adverse postoperative prognosis of patients with clear-cell renal cell carcinoma

**DOI:** 10.18632/oncotarget.12476

**Published:** 2016-10-05

**Authors:** Yang Qu, Li Liu, Jiajun Wang, Wei Xi, Yu Xia, Qi Bai, Ying Xiong, Qilai Long, Jiejie Xu, Jianming Guo

**Affiliations:** ^1^ Department of Urology, Zhongshan Hospital, Fudan University, Shanghai 200032, China; ^2^ Department of Biochemistry and Molecular Biology, School of Basic Medical Sciences, Fudan University, Shanghai 200032, China

**Keywords:** clear-cell renal cell carcinoma, Dot1l, overall survival, prognostic biomarker, nomogram

## Abstract

**Background:**

Disruptor of telomeric silencing 1-like (Dot1l), a histone methyltransferase that targets the histone H3 lysine 79 (H3K79), has been reported that its high expression is associated with various cancers, while the association between Dot1l expression and clear-cell renal cell carcinoma (ccRCC) is still unknown.

**Patients and Methods:**

We retrospectively enrolled 282 patients with ccRCC undergoing nephrectomy from a single institution between 2005 and 2007, with a median follow-up of 99 months. Dot1l expression was evaluated by immunohistochemistry in clinical specimens. We compared the clinical outcomes by Kaplan-Meier survival analyses and assessed the prognostic value of Dot1l expression. Harrell's concordance index (C-index) was used to assess the predictive accuracy of different prognostic models.

**Results:**

Higher Dot1l expression indicated poorer OS (*P*<0.001) and RFS (*P*<0.001) in patients with ccRCC. Moreover, Dot1l expression could stratify ccRCC patients in pT stage, Fuhrman grade and SSIGN/ Leibovich subgroups, which might redefine individual risk stratification. Multivariate analyses further indicated that Dot1l expression was an independent prognostic factor for OS (*P*=0.007) and RFS (*P*=0.001). The prognostic accuracy of conventional prognostic models was notably improved with Dot1l integration. Two nomograms and calibration plots were built to predict OS and RFS for patients with ccRCC and performed better based on C-index value.

**Conclusion:**

Dot1l expression is a promising independent prognostic indicator for postoperative recurrence and survival of patients with ccRCC.

## INTRODUCTION

Renal cell carcinoma (RCC), accounting for 2-3% of all human malignancies, is the most common cancer in kidney [1]. Among all histological subtypes of RCC, clear-cell renal cell carcinoma (ccRCC) is the commonest one accounting for more than 80% cases [2]. Although tremendous progress in diagnosis and treatment took place recently, radiography especially, it is still estimated that 62700 new cases and 14240 deaths will occur in the USA in 2016 [3]. Clinical outcomes are hard to predict for ccRCC because of heterogeneity of molecular phenotype [4]. Therefore, we need new and valuable prognostic biomarkers for ccRCC, besides of classical prognostic models, such as TNM stage, Fuhrman grade, Eastern Cooperative Oncology Group performance status (ECOG-PS), the Mayo clinic stage, size, grade, and necrosis score (SSIGN) [5] and the University of Los Angeles integrated staging system (UISS) [6] category systems. Furthermore, ccRCC has a high risk of metastasis (about 20-30%) and patients with metastasis usually get poor outcomes, which means biomarkers are needed for treatment, either [3].

Recently, post-translational histone modification has become more and more popular in cancer research due to their ability to regulate gene transcription. By modifying chromatin structure with methyl groups, histone methylation is the first identified post-translational histone modification among the four classic histone modifications [7]. In this process, the methyl groups attach to a lysine or arginine residue first, and connect to the -amine group with the help of proper methyltransferases [8]. Among all those modifications, methylation on histone H3 lysine 79 (H3K79) is a crucial one and has been studied recently. Furthermore, several researches revealed that methyltransferases and demethylases played key roles in the genesis and development of ccRCC, such as EZH2 and UTX [9].

Disruptor of telomeric silencing 1-like (Dot1l), located on the nucleosome surface, is the only known mammalian histone methyltransferase which targets the H3K79 position [10]. Just like its homolog gene Dot1 which first identified in yeast, Dot1l catalyzes the mono-, di-, and tri- methylation of H3K79 specifically with a unique catalytic domain. According to recent findings, Dot1l-mediated H3K79 methylation is associated with many biological processes including transcriptional regulation, DNA damage response, cell cycle progression, somatic reprogramming and embryonic cell development [11]. In addition, accumulating studies suggested that Dot1l plays an important role in the genesis and progression of mixed lineage leukemia (MLL) [12]. Still, several researches have shown that Dot1l takes part in the progression of many other tumors, such as lung cancer [13], colorectal cancer [14] and breast cancer [15]. As a promising biochemical target, one of the Dot1l inhibitors is already under investigation in a Phase I clinical trial in MLL patients [16].

However, the value of Dot1l in treatment and prognosis of ccRCC is still unknown as nobody studied on it. In this study, we analyzed the expression of Dot1l in ccRCC tissues by immunohistochemical analysis and dug out its association with clinicopathologic variables and clinical outcomes. Furthermore, we assessed the prognostic values of Dot1l expression and built two nomograms to predict individual risk for ccRCC patients.

## RESULTS

### Dot1l staining intensity

**Figure 1:**
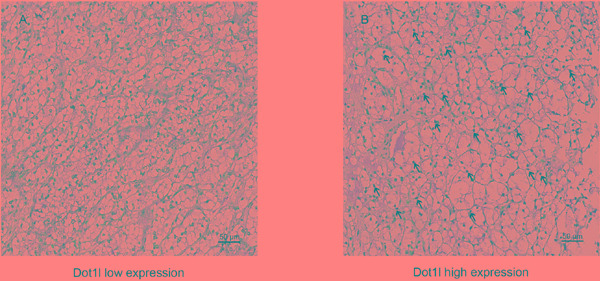
Dot1l expression in clear-cell renal cell carcinoma (ccRCC) tissues Representative Dot1l immunohistochemical images with low expression level **A.** and high expression level **B.** in ccRCC tissue at 200× optical magnification. Arrows indicate positive staining of Dot1l in each image. Scale bar: 50 μm.

In order to find out whether the Dot1l expression is connected with tumor characters, we evaluated the Dot1l expression by immunohistochemical staining analysis in all 282 patients’ samples first. As presented in Figure 1, Dot1l was predominantly expressed in the nucleus of tumor cells. As the intensity of specific staining was different in different cases, we defined the cutoff point as 95 (H-score range: 15-261) by X-tile software used minimum p value method.

### Patient characteristics and its association with tumoral Dot1l expression

As showed in Table 1, a total of 282 patients [196 male (69.5%) and 86 female (30.5%)] were enrolled in our study. The median follow-up was 99 months (range: 2.63-120.47 months). The median age of patients was 55 years with a range of 15–83 years, and the median tumor size was 4.8 cm with a range of 0.5–15.0 cm. The distribution of TNM stage at the time of surgery was I (62.1%), II (8.2%), III (23.0%), and IV (6.7%), respectively. Among all patients, 2 patients (0.7%) were in pN1 stage and 15 patients (5.3%) had been found distant metastases. Otherwise, the patients proportion of each Fuhrman grades was 1 (10.6%), 2 (73.8%), 3 (14.5%), and 4 (1.1%). In total, 13.8% cases were found necrosis at the surgery. Also, there were 27.0% patients with ECOG-PS ≥1. UISS category as low risk, mediate risk, and high risk were 42.9%, 47.5%, and 9.6 % of all cases, respectively. SSIGN category classified as 0-3, 4-7 and 8+ were 77.0%, 20.9% and 2.1% among all patients, respectively.

**Table 1:** Clinical characteristics of patients according to tumoral dot1l expression

Characteristics	Patients		Tumoral dot1l expression		
	n	%	low	high	P-value
All patients	282	100	147	135	
Age, years^#^					0.015^*^
mean±SD	55.2±12.9		53.4±12.7		57.1±12.8		
median	55		54		57		
range	15-83		15-81		27-83		
Gender					0.280†
Female	86	30.5	49	37	
Male	196	69.5	98	98	
Tumor size, cm^#^					0.251^*^
mean±SD	4.8±2.6		4.6±2.4		4.9±2.9		
Median	4.0		4.0		4.0		
range	0.5-15.0		0.5-13.0		1.0-15.0		
Pathological T stage					**0.006‡**
pT1	180	63.8	107	73	
pT2	28	10.0	9	19	
pT3	70	24.8	29	41	
pT4	4	1.4	2	2	
Pathological N stage					1.000†
pN0	280	99.3	146	134	
pN1	2	0.7	1	1	
Distant metastasis					**0.022†**
No	267	94.7	144	123	
Yes	15	5.3	3	12	
TNM stage					**0.001‡**
I	175	62.1	105	70	
II	23	8.2	8	15	
III	65	23.0	29	36	
IV	19	6.7	5	14	
Fuhrman grade					0.394‡
1	30	10.6	17	13	
2	208	73.8	109	99	
3	41	14.5	20	21	
4	3	1.1	1	2	
Necrosis					0.646†
Absent	243	86.2	128	115	
Present	39	13.8	19	20	
ECOG PS					0.131‡
0	206	73.0	113	93	
≥1	76	27.0	34	42	
UISS category					**0.006‡**
low risk	121	42.9	77	44	
mediate risk	134	47.5	57	77	
high risk	27	9.6	13	14	
SSIGN category					**0.001‡**
0-3	217	77.0	123	94	
4-7	59	20.9	24	35	
8+	6	2.1	0	6	

# Split at median;

* t-test for continuous variables,

† χtest or Fisher's exact test,

‡ Cochran-Mantel-Haenszel χtest, P-value<0.05 was regarded as statistically significant; ECOG PS, Eastern Cooperative Oncology Group performance status; UISS, UCLA Integrated Staging System; SSIGN, Mayo clinic stage, size, grade, and necrosis score.

Then, we analyzed the association of those clinicopathological variables with the Dot1l expression level. With the cutoff point of 95, we defined 147 (52.1%) patients with Dot1l low expression and 135 (47.9%) patients with a high expression level. Patients with higher Dot1lexpression trended to be older (*P*=0.015), have higher pT/pM stage (*P*=0.006/0.022) and have higher TNM grade (*P*=0.001). Also, UISS and SSIGN category were found statistically significant (*P*=0.006/0.001) with Dot1l expression level. The other clinicopathological characteristics had no statistically significant association with Dot1l expression level.

### High Dot1l expression is connected with dismal clinical outcomes of ccRCC patients

**Figure 2:**
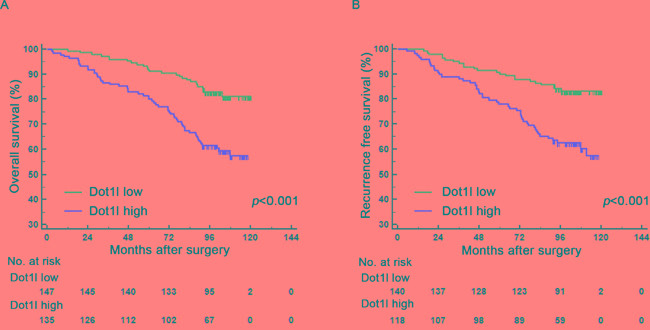
Kaplan-Meier analyses for overall survival (OS) and recurrence free survival (RFS) of patients with ccRCC based on Dot1l expression Kaplan-Meier analysis of OS in ccRCC patients (n=282) according to Dot1l expression **A.** Kaplan-Meier analysis of RFS in ccRCC patients (n=258) according to Dot1l expression **B.** *p*-value was calculated by Log rank test, *p*<0.05 was regarded as statistically significant.

We used Kaplan-Meier survival analysis to evaluate the clinical outcomes between different subgroups divided by Dot1l expression. As presented in Figure 2, patients in high Dot1l group had a worse OS (*P*<0.001, Figure 2A) and RFS (*P*<0.001, Figure 2B) than those in low Dot1l group. We further undertook univariate and multivariate analysis to make sure whether Dot1l is an independent predictor for ccRCC prognosis. As summarized in Table S1 Dot1l expression was strongly associated with clinical outcomes (OS, *P<*0.001; RFS, *P<*0.001) in univariate analysis. Also, pT/pM stage, Fuhrman grade, tumoral necrosis, tumor size and ECOG PS were each statistically significant in univariate analysis. We put those parameters in multivariate analysis and confirmed that Dot1l expression still was predictable for ccRCC outcomes (OS, *P=*0.007; RFS, *P=*0.001). Together with Dot1l, pT/pM stage, Fuhrman grade, tumoral necrosis and ECOG PS were all considered as independent predictors for ccRCC prognosis.

### Subgroup analysis for the relationship between Dot1l expression and clinical outcomes

**Figure 3:**
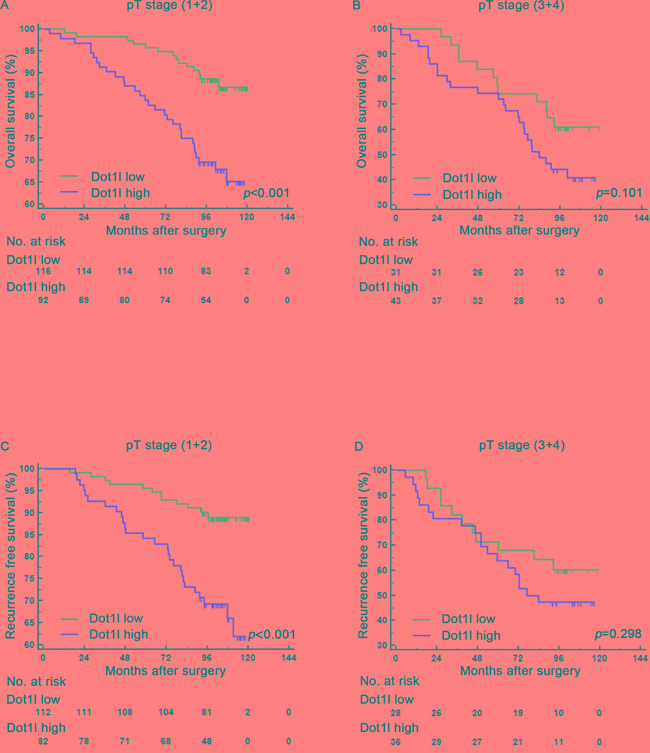
Subgroup analysis to assess prognostic value of Dot1l by pT stage in ccRCC patients Kaplan-Meier analysis of overall survival (OS) for patients in the pT stage (1+2) group **A.** and (3+4) group **B.** according to Dot1l expression; Kaplan-Meier analysis of recurrence free survival (RFS) for patients in the pT stage (1+2) group **C.** and (3+4) group **D.** according to Dot1l expression; *p*-value was calculated by Log rank test, *p*<0.05 was regarded as statistically significant.

To make sure whether the connection of Dot1l expression with clinical outcomes of patients depends on pT-stage, Fuhrman grade and SSIGN/Leibovich score, subgroup analysis of pT-stage, Fuhrman grade and SSIGN/ Leibovich were applied respectively. As presented in Figure 3, S1 and S2 both OS and RFS strongly associated with Dot1l expression in pT(1+2) group (*P*<0.001, Figure 3A, 3C), Fuhrman grade (1+2) group (*P*<0.001, *P*<0.001, Figure S1A, S1C) and SSIGN/Leibovich (0-3) group (*P*<0.001, Figure S2A, S2C). In the other hand, Dot1l failed to predict tumor outcomes in pT (3+4) group (*P*<0.001, Figure 3B, 3D), Fuhrman grade (3+4) group (*P*=0.079, *P*=0.113, Figure S1B, S1D) and SSIGN/Leibovich (≥4) group (*P*<0.001, Figure S2B, S2D).

### Comparison of the predictive ability between Dot1l expression and other prognostic models

In order to further acknowledge the predictive ability of Dot1l expression, we compared Dot1l expression with classic prognostic models, such as TNM stage, SSIGN and UISS scoring systems, respectively. Concordance index (*C*-index) and Akaike information criteria (AIC) analysis were used for prognostic power evaluation for each model. As presented in Table 2, the *C-*indexes of TNM, SSIGN, and UISS for OS were 0.717, 0.725, and 0.727, while Dot1l expression was 0.622. Noticeably, when Dot1l was added for OS, the *C-*index of those models was improved to 0.751, 0.752, and 0.733 respectively. More than this, the AIC value of each model combined with Dot1l was lower than it alone. Obviously, the *C-*index and AIC of RFS were in a similar way.

**Table 2:** Comparison of the predictive accuracy of the prognostic models

Models	Overall survival (n=282)		Recurrence free survival (n=258)^a^	
	C-index	*AIC*	C-index	*AIC*
Tumoral Dot1l	0.622	865.333	0.618	725.500
TNM stage	0.717	830.280	0.677	711.246
TNM stage + Tumoral Dot1l	0.751	822.778	0.720	702.452
SSIGN	0.725	834.666	0.707	704.077
SSIGN + Tumoral Dot1l	0.752	827.931	0.735	695.150
UISS	0.727	837.542	0.721	697.350
UISS + Tumoral Dot1l	0.733	827.688	0.761	687.820
Nomogram	0.803	785.920	0.797	654.507

C-index, concordance index; *AIC*, Akaike information criteria; SSIGN, Mayo clinic stage, size, grade, and necrosis score; UISS, UCLA Integrated Staging System. C-index were calculated from 1000 bootstrap samples to protect from overfitting.

a For recurrence-free survival analysis, 17 patients with metastasis ccRCC and 7 miss followed patients are excluded.

### Prognostic nomogram for OS and RFS in ccRCC patients

**Figure 4:**
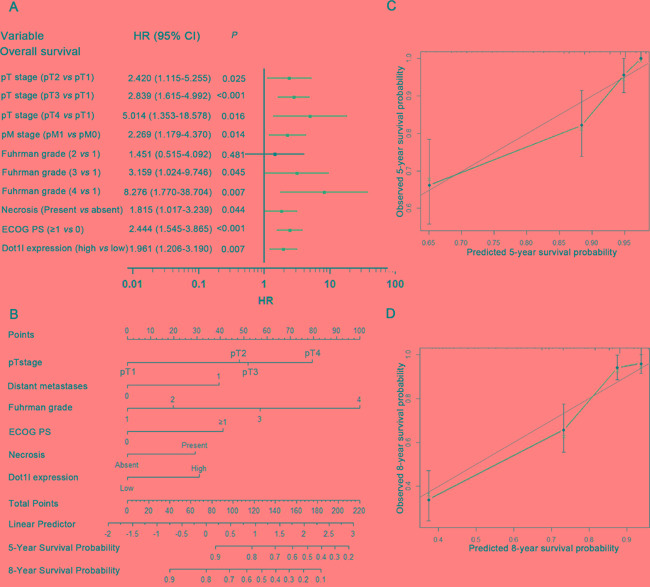
Multivariate analysis, Nomogram and calibration plots for prediction of overall survival (OS) in patients with ccRCC Multivariate analysis identified independent prognostic factors of OS **A.** nomogram to predict OS at 5- and 8- years after nephrectomy **B.** the calibration plots for predicting OS at 5-years **C.** and 8-years **D.**

In order to use Dot1l as a prognostic parameter, we build two nomograms for OS (Figure 4A, 4B) and RFS (Figure S3A, S3B) in ccRCC patients based on multivariate analysis. Total points were added from each point for each parameter, and could predict each patient's survival probability at different time after surgery. The calibration plots for the nomograms of OS and RFS presented good consistency between actual observation and the prediction by nomograms (Figure 4C, 4D; Figure S3C, S3D).

## DISCUSSION

To our knowledge, our study is the first paper reporting the association of Dot1l expression level and clinical outcomes of ccRCC patients among more than 5600 published reports about RCC prognostic markers. By multivariate analysis, our research confirmed that Dot1l could be regarded as an independent prognostic factor for ccRCC patients. Also, we found that patient with a higher Dot1l expression tends to have a worse clinical outcome in survival analyses. In the meanwhile, Dot1l expression could stratify ccRCC patients by survival analyses in pT stage (1+2), Fuhrman grade (1+2) and SSIGN (0-3) subgroups, which might redefine risk stratification of ccRCC patients. Furthermore, the incorporation of Dot1l expression into prognostic models such as SSIGN and UISS could dramatically enhance their prognostic accuracy. Finally, two nomograms were built to predict patients’ clinical outcomes based on Dot1l and other clinicopathologic parameters. It is nice to see that our nomograms have a better predictive performance than existed prognostic models based on C-index analysis.

It is well established that epigenetic modifications play a major role in genesis and development of cancer. Among those modifications, histone methylation acts as a key step because of its contribution to cell-cycle progression, somatic reprogramming and tumor genesis [11]. Dot1l, as a methyltransferase for histone H3K79 methylation, was found associations with several cancers. Barry E et al, demonstrated that Dot1l had a close connection with mixed lineage leukemia (MLL) and discussed several mechanisms [22]. In lung cancer, over expression of DOT1L leads to RNAi mediated changes which takes part in tumoral genesis [13]. In prostate cancer, Dot1l directly methylates androgen receptor to regulate its activity [23]. Besides, Zhang et al, showed that selective Dot1l inhibitors could suppress proliferation and migration in breast cancer cells [24]. Still, Dot1l has been reported to have association with other cancers, such as colorectal cancer [25]. However, the prognostic ability of Dot1l for ccRCC is still unknown. Moreover, we found that Dot1l copies had some mutations in ccRCC samples from the DNA information of 2013 TCGA cohort data, which indicated that Dot1l might be associated with ccRCC.

As the only known histone methyltransferase, which targets the histone H3K79, Dot1l adds methyl groups on histone H3K79 and generate mono- (H3K79me), di- (H3K79me2) and tri-methylation (H3K79me3).Up to now, several mechanisms have been summarized to explain the effect of Dot1l on tumor genesis and development. Firstly, Dot1l mediated methylation of H3K79 has been implicated in transcriptional elongation and cell cycle regulation, which influence the cell division and differentiation and then generate cancer cells. Schulze J et al, found that the level of H3K79me2 is different between different stages of cell cycle, while H3K79me3 remains constant throughout the cell cycle [26]. De Vos D et al, then demonstrated that H3K79me3level increases progressively in mutant cells [27]. Secondly, Dot1l has regulatory functions in gene transcription, which has a tight relationship with tumor genesis. An interesting phenomenon has been noticed that H3K79 mono- and di-methylation leads to activation of gene transcription, while H3K79 tri-methylation results in gene repression [11]. Thirdly, Dot1l has been proved to play a key role in haematopoiesis, and the high expression of Dot1l tends to cause MLL. GATA2, a growth factor essential for early haematopoiesis, was reported to be regulated by Dot1l and play a crucial role in this progress [28]. In addition, Dot1l has some other regulatory functions, which may have something to do with cancer, such as inhibition of somatic reprogramming [29] and promotion in DNA damage repair [11]. With those probable mechanisms, some Dot1l inhibitors were studied for anti-tumor treatment. Interestingly, Dot1l has a unique AdoMet binding motif for histone methylation, which is the only known non-SET domain histone methyltransferase protein [10]. This makes Dot1l to be a promising therapeutic target, and one of the Dot1l inhibitors is in phase I clinical trials [30].

However, the mechanism above cannot explain all tumors well as many downstream mechanisms are still unknown, especially for ccRCC. Our study focused on the connections between Dot1l expressions and the clinical outcomes of ccRCC patients, and confirmed the regulatory functions of Dot1l on tumor progression indirectly. According to our results, patients with early pT stage (1+2) could be stratified by Dot1l expression while those with late pT stage (3+4) could not. It is quiet similar in the SSIGN subgroup, which means that risk stratification of ccRCC patients might be redefined. Clinically, ccRCC patients with high Dot1l expression might need adjuvant therapy or a more proactive follow-up after surgery, even if they are low-risk patients based on classic clinicopathologic features. Furthermore, patients with better differentiation (Fuhrman grade 1+2) could be stratified by Dot1l expression, while those with higher Fuhrman grade (3+4) could not, which suggested that Dot1l could be involved in the tumor differentiation.

Although our study revealed the prognostic significance of Dot1l expression ccRCC patients, some limitations remain to be acknowledged. As this is a retrospective study and all samples and data of patients were collected from a single institution, a prospective, multicenter study is needed to further validate our results. Additionally, the proportion of patients with pT4 and pN1 were insufficient in our study, which might weaken Dot1l's predictive power in those subgroups. **Furthermore, the complete interaction network of Dot1l in ccRCC is still unclear and needs to be fully elucidated in future study, especially for the functional role of Dot1l in ccRCC.** Finally, future studies can focus on the therapeutic effect of Dot1l on ccRCC patients, as Dot1l inhibitors have been tested for MLL treatments

## CONCLUSION

In conclusion, our study indicated that Dot1l expression can be regarded as a prognostic factor for ccRCC patients. Patients with higher Dot1l expression tend to get poorer clinical outcomes. Dot1l expression could further stratify ccRCC patients in lower pT stage, Fuhrman grade and SSIGN category, which might redefine the risk stratification of ccRCC patients and guided clinical decisions. Last but not least, it is promising to explore Dot1l as a therapeutic target for ccRCC based on the crucial role of Dot1l in tumoral progression.

## MATERIALS AND METHODS

### Patients and database

A total of 282 patients with ccRCC who underwent nephrectomy were enrolled in base cohort retrospectively from the Department of Urology, Zhongshan Hospital, Fudan University (Shanghai, China) between Jan 2005 and Jun 2007. Clinical Research Ethics Committee of Fudan University approved the study with the approval number B2015-030 in Feb 2015 and each patient included in cohort was informed consent. The inclusion criteria for our study were conformed as follows: (a) confirmed postoperative histopathology diagnosis as ccRCC; (b) no adjuvant anticancer therapy after or before surgery and (c) no comorbidities. Patients who had histories of preoperative neoadjuvant and/or postoperative adjuvant therapy, perioperative mortalities, diagnosis of a mixture type of ccRCC and other RCC type were not included. For each patient, the following clinicopathological information was collected: age, gender, tumor size, pT stage, pN stage, pM stage, TNM stage, Fuhrman grade, presence of histological tumor necrosis, and ECOG PS. Each patient was staged with radiographic reports and postoperative pathological results, which were confirmed according to 2010 AJCC TNM classification [17]. Otherwise, ECOG PS score was calculated to each patient at the time of diagnosis. Both data were reassessed by two urologicpathologists (Yuan J. and H. Fu) independently using H&E-stained paraffin sections. Histological subtype of RCC was defined According to the 2014 EAU guidelines [18]. Fuhrman grade was confirmed according to 2012 ISUP consensus [19].The SSIGN, UISS score were applied to evaluated patient risks according to previous papers [5, 6].

Overall survival (OS) was calculated from the date of nephrectomy to the time of death or the most recent follow-up, while recurrence free survival (RFS) was calculated from the time of nephrectomy to the time of recurrence, which defined as local or distant metastases confirmed by imaging, biopsy or physical examination. Patients were followed-up with physical examination, laboratory studies, chest imaging and abdominal ultrasound or CT scan every 3 months for the first 5 years and annually thereafter. The last follow-up time was Jan 30, 2015. Totally 17 patients were excluded from RFS analysis for preoperational metastases and 7 patients for missing data of recurrence state. All baseline demographic, clinical, laboratory data, radiographic and pathological reports were reconfirmed by us.

### Immunohistochemistry and evaluation

Immunohistochemical staining was performed on tissue microarray (TMA) with antibody against Dot1l (ab64077, Abcam, 1:100 dilution) and proper visualization reagent (DakoEnVision Detection System) as previously described [20]. Olympus CDD camera, Nikon eclipse Ti-s microscope (×200 magnification) and NIS-Elements F3.2 software were used to record the results. The staining intensity and extent was scored by two independent pathologists without the knowledge of the patients’ outcomes. A semiquantitative H-score, ranged from 0 to 300, was used for each sample by evaluating the staining intensities (0: negative, 1: weak, 2: moderate, 3: strong) and distribution areas (0-100%). Three independent shots with strongest staining were selected for each core and the mean score of the three shots was regarded as the final staining intensity for each sample. The H-score cutoff point for determining tumoral Dot1l high/low expression is 95, which was evaluated by X-tile software (Yale University School of Medicine, New Haven, CT, USA) using minimum *p* value method [21].

### Statistical analysis

Statistical analysis in our study was performed by SPSS 21.0 (SPSS Inc., IL, Chicago, USA), MedCalc software (version 11.4.2.0; MedCalc, Mariakerke, Belgium), Stata 12.0 (StataCorp, College Station, TX, USA) and R software version 3.1.2 with the “rms” package (R Foundation for Statistical Computing, Vienna, Austria). χ2 test, Fisher's exact method test and Cochran-Mantel-Haenszel χ2 test were applied for categorical data, while Student's t test was used to analyze continuous variables, to assess the relationship between tumoral Dot1l expression and patients’ clinical parameters. Survival (including OS and RFS) curves were made using Kaplan-Meier method and log-rank test. Univariate and multivariate Cox proportional hazard models were used to find the impact of parameters on OS and RFS. Only those statistically significant parameters showed in the univariate analysis were considered in the multivariate analysis. Harrell's concordance index (C-index) and the Akaike information criterion (AIC) value were used to assess the predictive accuracy of different prognostic models. R software with “rms” package was used to generate the nomograms and calibration plots. Parameters which were statistical significant in multivariate analyses were selected to built nomograms. All statistical tests were two sided with a statistically significant difference considered at *P*<0.05.

## SUPPLEMENTARY MATERIALS FIGURES AND TABLES


