# Supplementary material for: Dot1l expression predicts adverse postoperative prognosis of patients with clear-cell renal cell carcinoma
Source: Oncotarget. 2016 Oct 5;7(51):84775–84. doi: 10.18632/oncotarget.12476 (PMC5356697; doi:10.18632/oncotarget.12476)
Supplement: Supplementary file 1 [file oncotarget-07-84775-s001.pdf]

Dot1l expression predicts adverse postoperative prognosis of patients with clear-cell renal cell carcinoma

SUPPLEMENTARY FIGURES AND TABLE

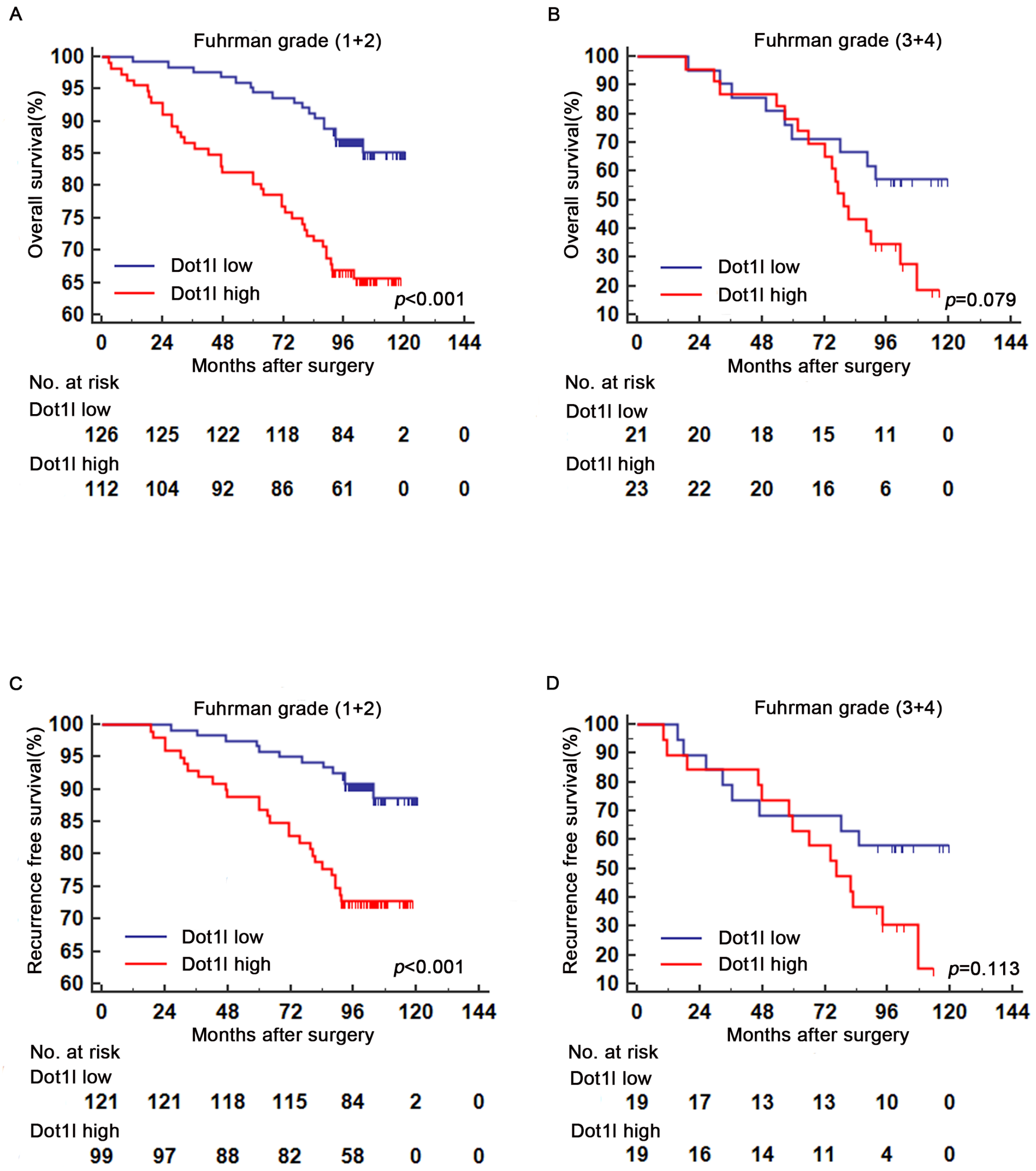

Supplementary Figure S1: Subgroup analysis to assess prognostic value of Dot1l by Fuhrman grade in ccRCC patients. Kaplan-Meier analysis of overall survival (OS) for patients in the Fuhrman grade (1+2) group **A.** and (3+4) group **B.** according to Dot1l expression; Kaplan-Meier analysis of recurrence free survival (RFS) for patients in the Fuhrman grade (1+2) group **C.** and (3+4) group **D.** according to Dot1l expression; *p*-value was calculated by Log rank test, $p<0.05$ was regarded as statistically significant.

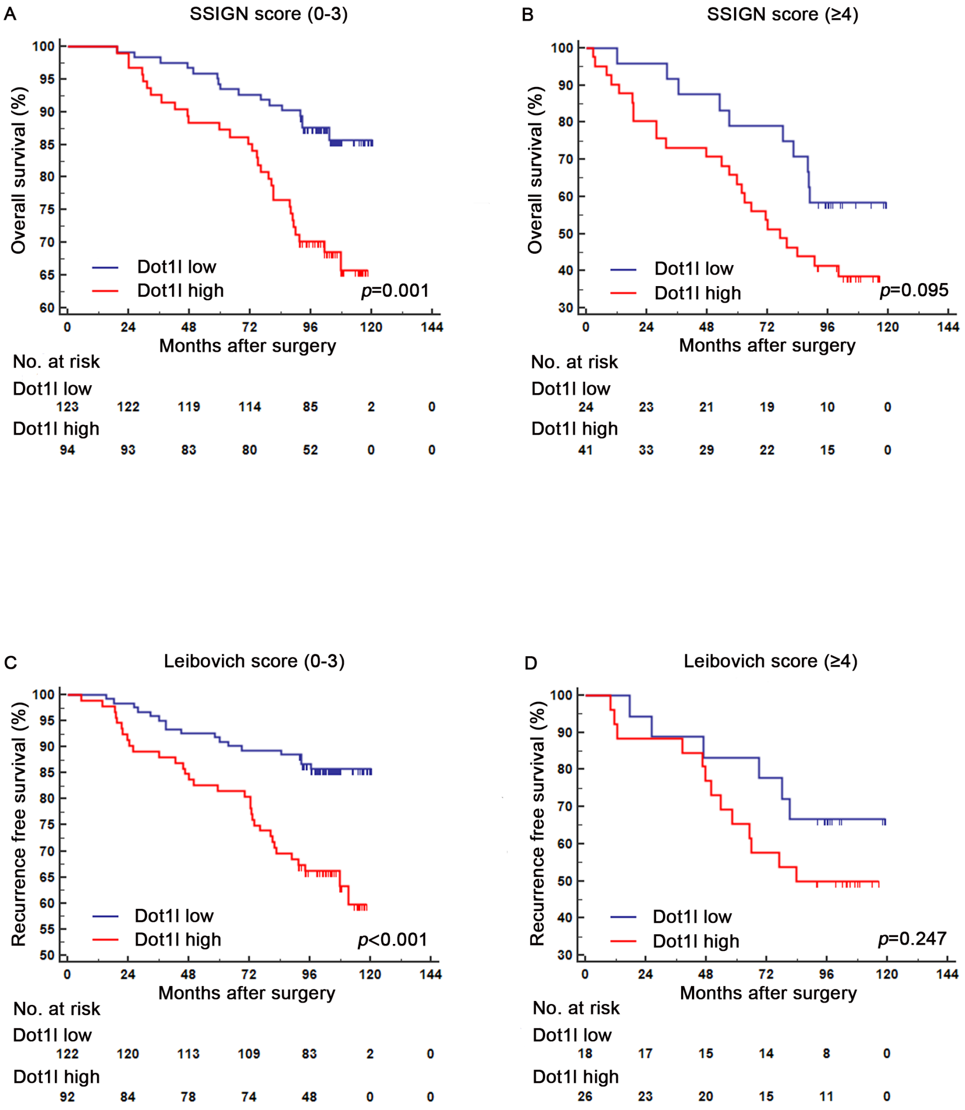

Supplementary Figure S2: Subgroup analysis to assess prognostic value of Dot1l by SSIGN/Leibovich category in ccRCC patients. Kaplan-Meier analysis of overall survival (OS) for patients in the SSIGN low risk (0-3) group **A.** and high risk (≥4) group **B.** according to Dot1l expression; Kaplan-Meier analysis of recurrence free survival (RFS) for patients in the Leibovich low risk (0-3) group **C.** and high risk (≥4) group **D.** according to Dot1l expression; *p*-value was calculated by Log rank test, $p<0.05$ was regarded as statistically significant.

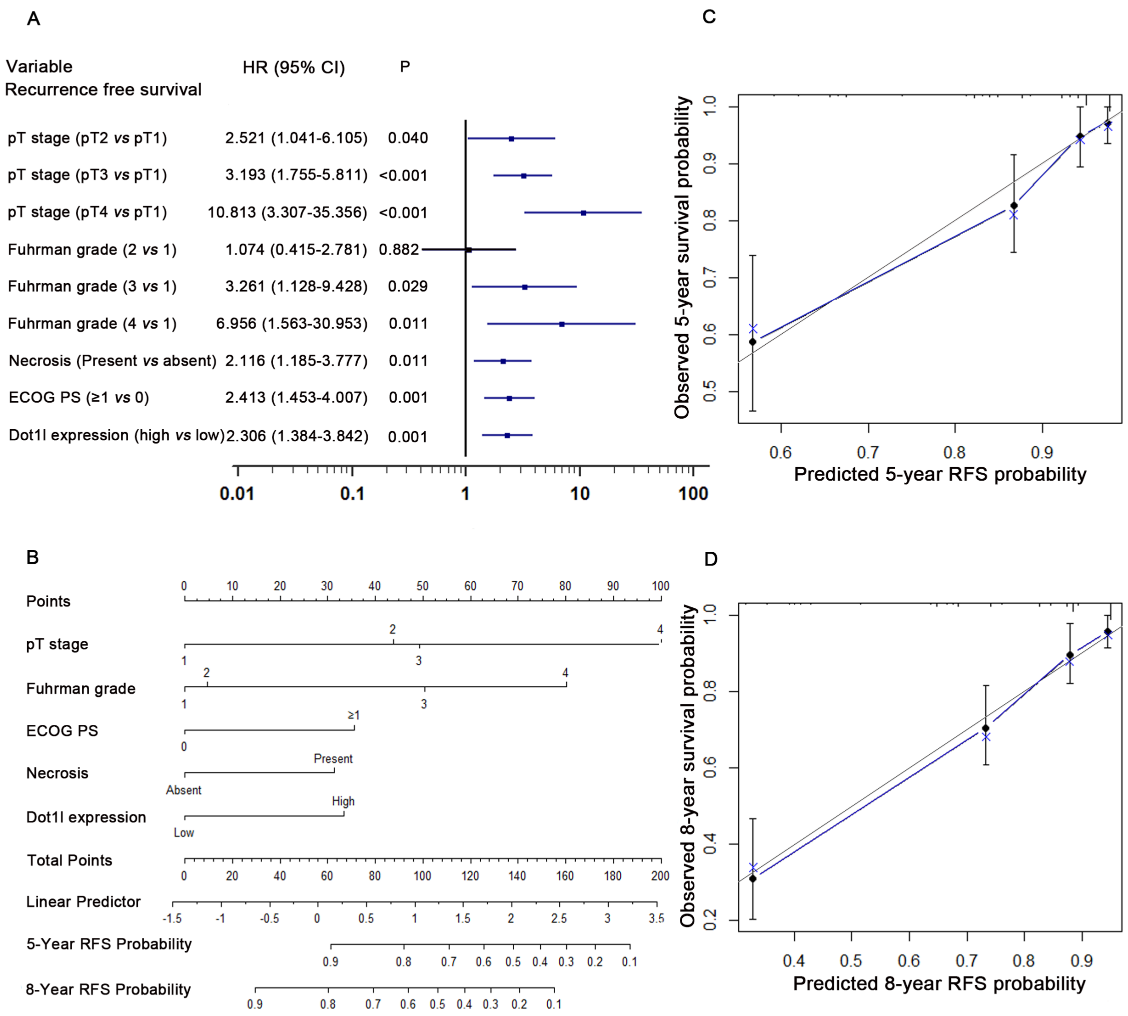

Supplementary Figure S3: Multivariate analysis, Nomogram and calibration plots for prediction of recurrence free survival (RFS) in patients with ccRCC. Multivariate analysis identified independent prognostic factors of RFS **A.** nomogram to predict RFS at 5- and 8- years after nephrectomy **B.** the calibration plots for predicting RFS at 5-years **C.** and 8-years **D.**

Supplementary Table S1: Univariate and multivariate cox regression analyses for overall survival (n=282) and recurrence free survival (n=258) in ccRCC patients

Variables	Univariate analysis		Multivariate analysis	
	HR(95% CI)	p^*	HR(95% CI)	p^*
**Overall survival**				
Gender (male *vs* female)	0.999 (0.623-1.601)	0.996	-	-
pT stage		**<0.001**		**0.002**
pT2 *vs* pT1	3.859 (2.044-7.287)	**<0.001**	2.420 (1.115-5.255)	**0.025**
pT3 *vs* pT1	3.778 (2.309-6.182)	**<0.001**	2.839 (1.615-4.992)	**<0.001**
pT4 *vs* pT1	8.106 (2.461-26.700)	**0.001**	5.014 (1.353-18.578)	**0.016**
pN stage (pN1*vs* pN0)	1.693 (0.235-12.180)	0.601	-	-
pM stage (pM1*vs* pM0)	6.221 (3.362-11.510)	**<0.001**	2.269 (1.179-4.370)	**0.014**
Fuhrman grade		**<0.001**		**0.002**
2 *vs* 1	1.997 (0.722-5.528)	0.183	1.451 (0.515-4.092)	0.481
3 *vs* 1	5.612 (1.938-16.254)	**0.001**	3.159 (1.024-9.746)	**0.045**
4 *vs* 1	7.862 (1.758-35.163)	**0.007**	8.276 (1.770-38.704)	**0.007**
Necrosis (present *vs* absent)	2.655 (1.613-4.370)	**<0.001**	1.815 (1.017-3.239)	**0.044**
Tumor size (continuous, cm)	1.198 (1.113-1.290)	**<0.001**	1.037 (0.940-1.145)	0.468
ECOG PS (≥1 *vs* 0)	3.382 (2.185-5.234)	**<0.001**	2.444 (1.545-3.865)	**<0.001**
Dot1l (high *vs* low)	2.682 (1.681-4.278)	**<0.001**	1.961 (1.206-3.190)	**0.007**
**Recurrence-free survival**				
Gender (male *vs* female)	0.894 (0.542-1.474)	0.660		
pT stage		**<0.001**		**<0.001**
pT2 *vs* pT1	4.037 (2.005-8.127)	**<0.001**	2.521 (1.041-6.105)	**0.040**
pT3 *vs* pT1	3.477 (2.037-5.937)	**<0.001**	3.193 (1.755-5.811)	**<0.001**
pT4 *vs* pT1	15.873 (5.453-46.205)	**<0.001**	10.813 (3.307-35.356)	**<0.001**
Fuhrman grade		**<0.001**		**<0.001**
2*vs* 1	1.351 (0.534-3.418)	0.526	1.074 (0.415-2.781)	0.882
3*vs* 1	4.301 (1.603-11.536)	**0.004**	3.261 (1.128-9.428)	**0.029**
4*vs* 1	6.250 (1.491-26.200)	**0.012**	6.956 (1.563-30.953)	**0.011**
Necrosis (present *vs* absent)	3.053 (1.798-5.185)	**<0.001**	2.116 (1.185-3.777)	**0.011**
Tumor size (continuous, cm)	1.184 (1.088-1.288)	**<0.001**	1.035 (0.925-1.157)	0.549
ECOG PS (≥1 *vs* 0)	3.085 (1.917-4.964)	**<0.001**	2.413 (1.453-4.007)	**0.001**
Dot1l (high *vs* low)	2.685 (1.627-4.432)	**<0.001**	2.306 (1.384-3.842)	**0.001**

Abbreviations: ECOG PS: Eastern Cooperative Oncology Group performance status; HR: hazard ratio; CI: confidence interval; ccRCC: clear cell renal cell carcinoma.

*Data obtained from the Cox proportional hazards model; $p<0.05$ was regard as statistically significant.
